# Longitudinal Analysis of Electronic Health Information to Identify Possible COVID-19 Sequelae

**DOI:** 10.3201/eid2902.220712

**Published:** 2023-02

**Authors:** Eleanor S. Click, Donald Malec, Jennifer R. Chevinsky, Guoyu Tao, Michael Melgar, Jennifer E. Giovanni, Adi V. Gundlapalli, S. Deblina Datta, Karen K. Wong

**Affiliations:** Centers for Disease Control and Prevention, Atlanta, Georgia, USA (E.S. Click, J.R. Chevinsky, G. Tao, M. Melgar, J.E. Giovanni, A.V. Gundlapalli, S.D. Datta, K.K. Wong);; Centers for Disease Control and Prevention, Hyattsville, Maryland, USA (D. Malec)

**Keywords:** COVID-19, coronavirus disease, severe acute respiratory syndrome coronavirus 2, SARS-CoV-2, coronaviruses, viruses, respiratory infections, longitudinal analysis, electronic health information sequelae, zoonoses, United States

## Abstract

Ongoing symptoms might follow acute COVID-19. Using electronic health information, we compared pre‒ and post‒COVID-19 diagnostic codes to identify symptoms that had higher encounter incidence in the post‒COVID-19 period as sequelae. This method can be used for hypothesis generation and ongoing monitoring of sequelae of COVID-19 and future emerging diseases.

SARS-CoV-2 causes acute COVID-19 and may cause post–COVID-19 conditions, which include a range of long-term sequelae (*1*,*2*). Review of multiple studies describes ongoing symptoms after acute COVID-19 (*3*). Post–COVID-19 conditions might include symptoms of nonspecific chest pain, fatigue, and malaise, as well as cardiomyopathy, renal failure, lung disease, and venous thromboembolism. Identifying possible sequelae of an emerging disease has traditionally required aggregating clinical experiences; this approach might miss sequelae that are rare or where the increase above baseline is not obvious (*4*).

Large electronic health information databases might aid in detecting these early signals, especially when potential sequelae events are temporally and geographically dispersed. The International Classification of Diseases, 10th revision (ICD-10), code for post‒COVID-19 conditions was not available for use in the United States until October 2021; thus, examining other diagnosis codes is needed to infer potential sequelae (*5*). We evaluated feasibility of a method comparing pre‒ and post‒COVID-19 diagnosis healthcare codes to identify possible sequelae from a large national database of healthcare encounters.

## The Study

The Premier Healthcare Database, Special COVID-19 Release (PHD-SR) is a large, hospital-based, service-level, all-payer database with >900 contributing hospitals and healthcare systems. The database includes diagnostic codes from the International Classification of Diseases, 10th Revision, Clinical Modification (ICD-10-CM), for inpatient and selected outpatient encounters with representation in all US census regions (*6*–*8*) (Appendix).

Using PHD-SR (release date February 4, 2021), we analyzed variables for encounter type (inpatient, outpatient including emergency), encounter date sequence variables (length of stay, admission and discharge month, and days between encounters), and discharge ICD-10-CM codes. We included patients with a first COVID-19 inpatient or outpatient encounter (i.e., a COVID-19 discharge ICD-10-CM code). COVID-19 index date was the first day of the first COVID-19 encounter. Pre‒COVID-19 encounters were any encounters within 365 days before the patient’s first COVID-19 encounter. Post‒COVID-19 encounters were the first COVID-19 encounter and all subsequent encounters.

We included encounters with discharge dates during January 1, 2019–December 31, 2020. We calculated relative rates (RRs) of post‒ to pre‒COVID-19 diagnoses in the post‒COVID-19 intervals of 60–89, 90–119, and 120–149 days, where day 0 is the COVID-19 index date. Rates were total number of encounters with a particular ICD-10-CM code observed in the specified time interval divided by total number of days in that interval that patients were also in the database (some patients might have died or been no longer observed in the database (i.e., right-censored). Pre-COVID-19 rates were calculated similarly for the whole interval, accounting for when they were first observed in the dataset (i.e., left-censored).

Because the day of reported diagnosis is only known to have occurred sometime between day of admission and day of discharge, we assigned a specific day (assigned randomly over the encounter duration) as the day of diagnosis for analysis. We generated 5 versions of the dataset with imputed diagnosis dates to capture this uncertainty. To compare diagnosis rates for the pre‒ and post‒COVID-19 intervals, we used 1-sided t-tests of the equality of rates performed on a log RR scale (*9*). We limited analyses to ICD-10-CM codes that occurred in >5 encounters during >1 post‒COVID-19 interval because of difficulty in interpreting RR for rare events (Appendix).

We evaluated whether RR was >1 in the post‒ versus pre‒COVID-19 intervals by using a t-test that includes variability due to multiple imputations (*10*). We report results significant at p<0.05 after performing the Benjamini-Hochberg adjustment procedure that excludes marginally significant results that could have occurred by chance because of performing a large number of significance tests. We performed analyses in R 3.6.0 (The R Foundation for Statistical Computing, https://cran.r-project.org/bin/windows/base/old/3.6.0). We defined diagnoses with significantly increased encounter rates >60 days after COVID-19 index date relative to pre–COVID-19 as possible postacute sequelae. 

We identified 385,067 patients with a COVID-19 discharge date January–December 2020 and >1 visit within the previous 365 days. Median encounters per patient was 4 (interquartile range [IQR] 3–7; pre–COVID-19, 2 [IQR 1–5]; post–COVID-19, 1 [IQR 1–2]); 87% were outpatient encounters. The cohort was 59% female. Median age was 54 (IQR 35–69) years; 5.1% were <18 years of age. Median length of stay for inpatient encounters was 4 (IQR 2–8) days.

Encounters for sequelae of specified infectious and parasitic diseases were increased at least through 149 days after the index date (RR 11.6 at 120–149 days) (Table). Encounters were increased for several months after acute illness for postviral fatigue syndrome, headache, and certain respiratory diseases, including pneumonia and acute respiratory distress syndrome. We identified general sequelae of treatment in intensive care, including polyneuropathy (RR 9.1 at 90–119 days) and myopathy (RR 5.0 at 60–89 days), nonscarring hair loss (RR 2.3–3.5 in multiple intervals beyond 60 days), and pressure ulcers (stage 3 and 4, RR 1.6–1.7 at 60–89 days).

**Table:** Diagnostic codes reported during post–COVID-19 interval with increased rate >60 d after acute illness relative to pre–COVID-19 baseline rate, by interval from index date, from large administrative all-payer database, United States, January 2019–December 2020*

Diseases and codes	Rate increase relative to pre–COVID-19 rate (SE)†				Post–COVID-19 rate, encounters/1,000 person-months		
	60–89 d	90–119 d	120–149 d		60–89 d	90–119 d	120–149 d
Certain infectious and parasitic diseases							
B97.21: SARS-associated coronavirus as the cause of diseases classified elsewhere	28.7 (12.8)	31.9 (15.6)	82.2 (39.5)		0.22	0.24	0.63
B94.8: Sequelae of other specified infectious and parasitic diseases	15.4 (4.5)	17 (5.1)	11.6 (4.2)		0.26	0.29	0.20
A41.53: Sepsis due to *Serratia*	6 (3.9)	NS	NS		0.60	NS	NS
A41.89: Other specified sepsis	2.8 (0.5)	NS	NS		0.69	NS	NS
B34.2: Coronavirus infection, unspecified	2 (0.6)	2.4 (0.7)	NS		0.24	0.29	NS
R65.21: Severe sepsis with septic shock	1.5 (0.2)	NS	NS		1.29	NS	NS
Diseases of blood and blood-forming organs and certain disorders involving the immune mechanism							
D84.821: Immunodeficiency due to drugs	7.9 (6.0)	NS	NS		0.30	NS	NS
Diseases of the circulatory system							
B33.24: Viral cardiomyopathy	9.8 (8.8)	NS	NS		0.30	NS	NS
I46.8: Cardiac arrest due to other underlying condition	4.8 (2.3)	3.5 (1.9)	NS		0.70	0.50	NS
I46.9: Cardiac arrest, cause unspecified	4.6 (0.9)	3.7 (0.9)	4.5 (1.1)		0.40	0.32	0.38
I40.0: Infective myocarditis	NS	12.6 (10.6)	NS		NS	0.40	NS
Diseases of the digestive system							
K20.91: Esophagitis, unspecified with bleeding	19.8 (18.4)	NS	NS		0.30	NS	NS
K21.00: Gastro-esophageal reflux disease with esophagitis, without bleeding	5.7 (1.3)	6.1 (1.5)	5.7 (1.7)		0.29	0.31	0.29
K20.90: Esophagitis, unspecified without bleeding	3.1 (1.4)	5.8 (2.3)	NS		0.70	0.13	NS
K00.7: Teething syndrome	NS	4 (2.3)	NS		NS	0.50	NS
Diseases of the genitourinary system							
N18.31: Chronic kidney disease, stage 3a	4 (1.4)	5.1 (1.9)	6.4 (2.2)		0.14	0.18	0.22
N18.30: Chronic kidney disease, stage 3 unspecified	3.6 (0.3)	3.8 (0.4)	4.2 (0.5)		1.83	1.91	2.13
N18.32: Chronic kidney disease, stage 3b	2.5 (1.0)	3.2 (1.2)	5.1 (1.9)		0.90	0.12	0.19
Diseases of the musculoskeletal system and connective tissue							
M62.59: Muscle wasting and atrophy, not elsewhere classified, multiple sites	NS	4.1 (2.1)	NS		NS	0.10	NS
M65.071: Abscess of tendon sheath, right ankle and foot	NS	NS	280.3 (550.3)		NS	NS	0.10
Diseases of the nervous system							
G62.81: Critical illness polyneuropathy	11.8 (6.8)	9.1 (6.8)	NS		0.70	0.50	NS
G93.3: Postviral fatigue syndrome	6.8 (2.5)	4.2 (2.1)	NS		0.12	0.70	NS
G72.81: Critical illness myopathy	5 (1.5)	NS	NS		0.30	NS	NS
R51.9: Headache, unspecified	2.6 (0.3)	3.9 (0.3)	3.9 (0.4)		1.47	2.23	2.23
Diseases of the respiratory system							
J12.81: Pneumonia due to SARS-associated coronavirus	8.5 (3.6)	NS	NS		0.90	NS	NS
J80: Acute respiratory distress syndrome	7.4 (2.9)	3.9 (1.7)	NS		0.32	0.17	NS
J95.851: Ventilator associated pneumonia	5.2 (2.4)	4 (2.1)	NS		0.90	0.70	NS
J12.89: Other viral pneumonia	NS	16.1 (2.6)	11 (2.0)		NS	1.33	0.91
Diseases of skin and subcutaneous tissue							
L65.9: Nonscarring hair loss, unspecified	2.4 (0.5)	3.5 (0.7)	2.3 (0.6)		0.36	0.54	0.35
L89.153: Pressure ulcer of sacral region, stage 3	1.7 (0.4)	NS	NS		0.42	NS	NS
L89.154: Pressure ulcer of sacral region, stage 4	1.6 (0.3)	NS	NS		0.68	NS	NS
Endocrine, nutritional, and metabolic diseases							
E87.71: Transfusion associated circulatory overload	4.9 (3.0)	NS	NS		0.40	NS	NS
Mental, behavioral, and neurodevelopmental disorders							
F10.139: Alcohol abuse with withdrawal, unspecified	8.8 (7.6)	NS	NS		0.30	NS	NS
Neoplasms							
C83.90: Non-follicular (diffuse) lymphoma, unspecified, unspecified site	411 (807)	272.6 (535.2)	NS		0.14	0.90	NS
Symptoms, signs, and abnormal clinical and laboratory findings, not elsewhere classified							
R74.01: Elevation of liver transaminase levels	4.8 (1.2)	5 (1.4)	6.5 (1.9)		0.21	0.21	0.28

*Results are significant at p<0.05. Diagnoses are limited to those with RRs significantly increased at >60 d. Clinical Classification Software Refined categories were taken from Agency for Healthcare Research and Quality Clinical Classifications Software Refined (https://www.hcup-us.ahrq.gov/toolssoftware/ccsr/ccs_refined.jsp). NS, not significant; RR, relative rate. †A specific diagnosis is counted for each patient encounter so that 1 patient might contribute >1 diagnosis if they were admitted for that diagnosis more than once during time period of interest.

Viral cardiomyopathy (RR 9.8 at 60–89 days) and sepsis codes were only increased in the first 90 days after index date. Rates of nonfollicular diffuse lymphomas were increased in the 60–119 day periods (RR 272.6–411), but most encounters were for 1 patient. Encounters for stage 3 chronic kidney disease (RR 2.5–6.4 beyond 60 days) and for increased liver aminotransferase levels (RR 4.8–6.5 beyond 60 days) were higher for several months after the index date; infective myocarditis (RR 12.6) was increased for 90–119 days.

The possible cardiac, respiratory, kidney, and liver sequelae identified through this method are consistent with those of previous studies (*11*–*13*). For kidney injury, new diagnoses of stage 3 kidney illness (glomerular filtration rate 30–59 mL/min/1.73 m^2^) were higher than pre–COVID-19. Stage 3 kidney injury might occur when there is more permanent damage requiring repeated healthcare encounters. This method might generate useful hypotheses about the duration of possible sequelae because we found that encounters for increased aminotransferase levels remain increased at least through the 120–149-day interval after acute illness.

The first limitation of our study is that increased encounter rates might be caused by health-seeking behavior. Encounters for new diagnoses are not equivalent to new disease entities, and rates of encounter diagnosis codes might not represent the rates of disease. For long hospitalizations, diagnosis timing might be mischaracterized because actual diagnosis date is uncertain; however, we used imputation to account for this uncertainty. Counting the initial COVID-19 visit as part of the post–COVID-19 period might identify complications of acute illness as sequelae; however, we focused on sequelae with increased rates >60 days after the COVID-19 index date to mitigate that factor. This analysis does not capture exacerbations of underlying conditions, such as worsening heart failure or reactive airway disease, unless disease exacerbation is captured by a different diagnosis code.

The data in this analysis are more representative of adults than children. We excluded pregnancy-related conditions from the analysis. Our findings might not be generalizable to patients with asymptomatic or mild COVID-19 who might not seek healthcare, and we did not control for factors such as aging and changes in societal behavior, so we cannot attribute increased rates of new diagnoses solely to COVID-19. Advantages of our method include rapid application to large longitudinal healthcare datasets and extension over time to identify possible sequelae occurring long after acute illness. 

## Conclusions

Our findings are consistent with those of other studies using different methods to identify sequelae, including a matched cohort analysis of PHD-SR during the same period and a direct survey of persons with and without previous SARS-CoV-2 test results (*14*,*15*). This hypothesis-generating method can provide early signals of possible sequelae for novel diseases and inform additional studies to identify, characterize, and refine potential sequelae for COVID-19 or other emerging diseases.

Appendix Additional information on longitudinal analysis of electronic health information to identify possible COVID-19 sequelae.
