# Supplementary material for: Longitudinal Analysis of Electronic Health Information to Identify Possible COVID-19 Sequelae
Source: Emerg Infect Dis. 2023 Feb;29(2):389–92. doi: 10.3201/eid2902.220712 (PMC9881771; doi:10.3201/eid2902.220712)
Supplement: Appendix — Additional information on longitudinal analysis of electronic health information to identify possible COVID-19 sequelae. [file 22-0712-Techapp-s1.pdf]

Article DOI: https://doi.org/10.3201/eid2902.220712

Longitudinal Analysis of Electronic Health Information to Identify Possible COVID-19 Sequelae

Appendix

The study proposal was reviewed by CDC and conducted consistent with applicable federal law and CDC policy (e.g., 45 C.F.R. part 46.102(l) (*2*), 21 C.F.R. part 56; 42 U.S.C. §241(d); 5 U.S.C. §552a; 44 U.S.C. §3501 et seq).

1) U07.1 or 2) B97.29 (other coronavirus as the cause of diseases classified elsewhere) were restricted to admission months February–April 2020 and discharge months March–December 2020 during January–December 2020 with ≥1 encounter before first COVID-19 encounter.

We excluded codes in the following categories because of the complexity of defining an appropriate baseline rate for comparison: Certain Conditions Originating in the Perinatal Period and Pregnancy, Childbirth, and the Puerperium. Routine pregnancy-related codes are less likely to be observed in the pre-COVID period, because those who gave birth in a pre-COVID period are often young and otherwise healthy, and they are less likely to be included in the cohort which is defined by medically attended COVID-19. We excluded codes under Congenital Malformations, Deformations, and Chromosomal Abnormalities, because these are conditions that almost certainly precede the COVID-19 illness, even if they were first diagnosed or recorded after the acute illness. We excluded codes under Factors Influencing Health Status and Contact with Health Services because conditions that might generate these codes are also likely to generate more specific diagnosis codes in other categories as well as Injury, Poisoning and Certain Other Consequences of External Causes (*1*).

Reference

1. Chevinsky JR, Tao G, Lavery AM, Kukielka EA, Click ES, Malec D, et al. Late conditions diagnosed 1–4 months following an initial coronavirus disease 2019 (COVID-19) encounter: a matched-cohort study using inpatient and outpatient administrative data-United States, 1 March-30 June 2020. Clin Infect Dis. 2021;73(Suppl 1):S5–16. PubMed https://doi.org/10.1093/cid/ciab338
